# Multifunctional Nanoparticles Based on Iron Oxide and Gold-198 Designed for Magnetic Hyperthermia and Radionuclide Therapy as a Potential Tool for Combined HER2-Positive Cancer Treatment

**DOI:** 10.3390/pharmaceutics14081680

**Published:** 2022-08-12

**Authors:** Michał Żuk, Rafał Podgórski, Anna Ruszczyńska, Tomasz Ciach, Agnieszka Majkowska-Pilip, Aleksander Bilewicz, Paweł Krysiński

**Affiliations:** 1 Faculty of Chemistry, University of Warsaw, Pasteura 1 Str., 02-093 Warsaw, Poland; 2 Faculty of Chemical and Process Engineering, Warsaw University of Technology, Warynskiego 1 Str., 00-645 Warsaw, Poland; 3 Biological and Chemical Research Centre, Faculty of Chemistry, University of Warsaw, Zwirki i Wigury 101 Str., 02-089 Warsaw, Poland; 4 Institute of Nuclear Chemistry and Technology, Dorodna 16 Str., 03-195 Warsaw, Poland

**Keywords:** nuclear medicine, magnetic hyperthermia, targeted therapy

## Abstract

Iron oxide nanoparticles are commonly used in many medical applications as they can be easily modified, have a high surface-to-volume ratio, and are biocompatible and biodegradable. This study was performed to synthesize nanoparticles designed for multimodal HER2-positive cancer treatment involving radionuclide therapy and magnetic hyperthermia. The magnetic core (Fe_3_O_4_) was coated with a gold-198 layer creating so-called core-shell nanoparticles. These were then further modified with a bifunctional PEG linker and monoclonal antibody to achieve the targeted therapy. Monoclonal antibody—trastuzumab was used to target specific breast and nipple HER2-positive cancer cells. The nanoparticles measured by transmission electron microscopy were as small as 9 nm. The bioconjugation of trastuzumab was confirmed by two separate methods: thermogravimetric analysis and iodine-131 labeling. Synthesized nanoparticles showed that they are good heat mediators in an alternating magnetic field and exhibit great specific binding and internalization capabilities towards the SKOV-3 (HER2 positive) cancer cell line. Radioactive nanoparticles also exhibit capabilities regarding spheroid degradation without and with the application of magnetic hyperthermia with a greater impact in the case of the latter. Designed radiobioconjugate shows great promise and has great potential for in vivo studies regarding magnetic hyperthermia and radionuclide combined therapy.

## 1. Introduction

Iron oxide nanoparticles (Fe_3_O_4_) especially their superparamagnetic variation (SPION) paved the way for use of nanoparticles in the broad medical field: Feraheme^®^, Feridex^®^ and GastroMARK^®^ are just a few of the registered medical products [1,2,3]. After many years of research, these nanoparticles still find innovative use in many research fields, e.g., drug delivery [4,5,6], magnetic resonance imaging (MRI) [7,8], gene magnetofection [9,10], magnetic hyperthermia [11,12], and radionuclide therapy [13,14,15]. The last two are under our group’s particular attention. Thanks to the heat generation capabilities of iron oxide nanoparticles under alternating magnetic field (AMF) and ease in doping with radionuclide elements these structures show great promise for use in combination of radionuclide therapy with magnetic hyperthermia.

Targeted radionuclide therapy involves a radioactive drug called a radiopharmaceutical that targets cancer cells. Radiopharmaceuticals typically consist of a radionuclide attached to a cell-targeting molecule that binds to the cell surface receptors and destroys cancer cells. Therapeutic radiopharmaceuticals are mostly radionuclide complexes attached to the biomolecules, such as peptides (e.g., ^177^Lu-DOTATATE) or antibodies (e.g., ^90^Y-anti-CD20 monoclonal antibody). The radionuclide selection criteria for targeted radionuclide therapy depend on the physical parameters of the radionuclide (half-life, type of emitted particles, gamma ray emission, etc.), production method, separation chemistry, labeling chemistry and biological behavior. Currently, many radionuclides are under intensive investigation for the therapeutic applications, particularly the Auger emitters ^67^Ga (*t*_1/2_ = 3.3 d), ^111^In (*t*_1/2_ = 2.8 d), *β*-particle emitters ^90^Y (*t*_1/2_ = 64 h), ^188^Re (*t*_1/2_ = 16.7 h), ^177^Lu (*t*_1/2_ = 6.7 d), ^161^Tb (*t*_1/2_ = 6.9 d), ^166^Ho (*t*_1/2_ = 26.8 h) and α particle emitters ^211^At (*t*_1/2_ = 7.2 h), ^225^Ac (*t*_1/2_ = 10 d), ^213^Bi (*t*_1/2_ = 45.6 m). An important factor is the nature of the cytotoxic radionuclide, mainly its physical parameters, such as half-life and the nature of its associated emissions. Solid tumors generally have been pursued with high and medium energy *β*^−^ emitters including ^90^Y, ^188^Re,^131^I and ^166^Ho because their *β*^−^ particles have a tissue range of several millimeters. The effective tissue range of *β*^−^ particles is not optimal for the treatment of tumors as small clusters of cells or single cells, and micrometastases, because much of the decay energy is deposited outside the boundary of the tumor. For example, ^90^Y has a maximum range on the order of several hundred cell diameters and thus deposits energy in the tumor as well as the surrounding normal tissue. Treatment of these diseases might be more effective with low energy *β*^−^ emitters, which combine short range and high linear energy transfer, a combination that results in the high relative biological effect and cytotoxicity [16].

Hyperthermia is when elevating the temperature in tumor localization can damage, impair, or even kill malignant cells. The introduction of hyperthermia into clinical practice has been limited due to the inability to generate localized temperature increases solely in the tumor without influencing surrounding tissues. Recently this problem has vanished due to the use and development of modified iron oxide nanoparticles [17,18,19]. This so-called magnetic hyperthermia can destroy cancer cells by temperature increase that is precisely controlled and limited to malignant tissue [20,21]. NanoTherm^®^ was one of the first therapies approved to use in clinical practice by the FDA to treat glioblastoma multiforme and is in a clinical trial for prostate cancer [22,23]. Mild hyperthermic treatment can be effectively combined with other antitumor therapies, e.g., chemo-, endoradio-, and photodynamic therapy with greater therapeutic effect [24,25,26].

External ionizing radiation combined with hyperthermia could increase the efficiency of radiation in cancer treatment. This could contribute to achieving better results with the same dose of radiation or decrease the dose delivered without lowering the treatment yield [27]. The biological basis for this effect is that mild hyperthermia causes an increase in intratumoral blood flow, thus subsequent re-oxygenation facilitates the generation of reactive oxygen species (ROS) by ionizing radiation [27]. This leads to an increase in DNA damage. A similar effect should be achieved in magnetic hyperthermia combined with radionuclide therapy. Several publications describe the therapeutic effect achieved by the use of radionuclide-labeled magnetic iron oxide nanoparticles. M. Ognjanovic et al. and A. Stanković et al. [28], applied ^99m^Tc, ^90^Y, ^177^Lu and ^131^I labeled iron oxide nanoflowers to combine magnetic hyperthermia with radionuclide cancer therapy and diagnosis [28,29]. Additionally, in our previous works we investigated the possibility of using SPION and barium ferrite nanoparticles labeled with radionuclides ^166^Ho, ^161^Tb ^223^Ra and ^225^Ac for simultaneous radionuclide therapy and hyperthermia [15,30,31]. Unfortunately, direct labeling of SPION and barium ferrite nanoparticles did not allow obtaining the appropriate activities for therapy specific. There was also significant leakage of radionuclides from the labeled nanoparticles.

Due to the ease of modification of SPION by gold layer, in our work, we propose the gold-198 for labeling magnetic nanoparticles. ^198^Au can be obtained with good specific activity by thermal neutron irradiation of the monoisotopic target ^197^Au. The high cross-section for ^197^Au (n,γ) ^198^Au nuclear reaction (98.7 barn) allows us to receive 16 GBq of ^198^Au (1 mg Au target, 10^14^ n/cm^2^/s thermal neutron flux, 70 h irradiation, reactor Maria, Świerk, Poland). Gold complexes of Au^+^ and Au^3+^ have not yet been used in radionuclide therapy, because of their low stability in vivo. Upon exposure to the enzymes, the gold cations usually lose their former ligands and are coordinated by cysteine residues in the active site of glutathione reductase [32]. ^198^Au is a low energy β^−^ emitter due to its nuclear properties (T_1/2_ = 2.7 d, β_max_ = 0.96 MeV) and might be used in radionuclide therapy of small inoperable tumors. Thus, applying gold-198 emitting ionizing radiation as a shell on iron oxide delivering hyperthermia could make this multimodal platform for combination therapy possible.

In this paper, we present a new idea for combining radionuclide therapy with magnetic hyperthermia through the application of core-shell superparamagnetic Fe_3_O_4_ covered with a ^198^Au layer and conjugated with trastuzumab (Tmab)—monoclonal antibody used to target the HER2 positive cancer cells. Obtained radiobioconjugates were tested for stability in storage media, cytotoxicity, influence on spheroids degradation, binding affinity, and internalization. Physiochemical characteristics included magnetic hyperthermia studies, microscopic analysis, and dynamic light scattering studies.

## 2. Materials and Methods

### 2.1. Materials

Iron (III) chloride hexahydrate (97%), iron (II) chloride tetrahydrate (≥99%), triammonium citrate dihydrate (≥97.0%), glycine (≥99.0%), bifunctional HS-PEG-COOH, 5 kDa were purchased from Sigma-Aldrich (St. Louis, MO, USA). Sodium hydroxide (≥99.9%), and 25% ammonia solution was supplied from POCH (POCH, Wrocław, Poland). Bifunctional HS-PEG-NHS ester, 5 kDa was purchased from NANOCS (PEGLAB, Boston, MA, USA). Trastuzumab (Herceptin^®^) was supplied from Roche (Roche Pharma AG, Grenzach-Wyhlen, Germany). PBS 10× buffer pH 7.4 was purchased from VWR Life Science (Randor, PA, USA). MTS was supplied from Promega (Promega, Madison, WI, USA). PD10 columns filled with Sephadex G-25 resin were bought from GE Healthcare Life Sciences (Piscataway, NJ, USA). Gold-198 was obtained by neutron irradiation of a solid gold-197 target (99.99%) at research reactor Maria NCNR (National Center for Nuclear Research, Otwock-Świerk, Poland). Water DI (ultrapure, resistivity 18.2 MΩ·cm at 25 °C) was obtained on site using the Milli-Q^®^ ultrapure water filtering system from Merck-Millipore.

The SKOV-3 cell line was purchased from the ATCC American Type Culture Collection (American Type Culture Collection, Rockville, MD, USA) and cultivated in McCoy’s 5A medium. The growth medium was fortified with 10% heat-inactivated FBS (fetal bovine serum) and Pen-Strep: streptomycin (100 μg/mL), and penicillin (100 IU/mL). Cells were cultivated in a humidified atmosphere of 5% CO_2_ at 37 °C according to the manufacturer’s protocol. For the experiment, the cells were detached using 0.25% trypsin–EDTA solution. Reagents for cell growth were purchased from Biological Industries (Beth Haemek, Israel). For statistical analysis, GraphPad Prism 9 program was used (Dotmatics, San Diego, CA, USA).

### 2.2. Synthesis and Modification NPs Core

The synthesis of the Fe_3_O_4_ core was performed with a co-precipitation technique based on our previous work, but without the incorporation of holmium ions into the NPs structure [30]. Briefly, Fe_3_O_4_ nanoparticles were synthesized by mixing 0.2 M Fe^3+^ and 0.1 M Fe^2+^ solutions in a 2:1 volume ratio and co-precipitation with 25% NH_3(aq)_ solution (1450 rpm, 95 °C) for 15 min. To stabilize the core, 300 μL of 0.456 M triammonium citrate dihydrate (CA) was added. The reaction was carried out for an additional 30 min with mixing and heating at previous settings. The product was separated with magnetic sedimentation, and washed two times with cold acetone.

### 2.3. Synthesis of Fe_3_O_4_@Au Core-Shell NPs

To synthesize the gold-198 shell around the citrate stabilized Fe_3_O_4_ core, a modified method published by H. Zhou et al. was used [33,34]. In a round bottom flask containing boiling (under reflux) c.a. ^198^Au/Au solution (pH set to 6–7), the nanoparticle’s core suspension was added. The coating reaction was allowed to continue for 30 min with moderate stirring (600 rpm). The product was purified by magnetic sedimentation and washed several times with DI water.

### 2.4. Synthesis of Core-Shell Radiobioconjugates

NPs obtained previously were mixed with HS-PEG-NHS ester/HS-PEG-COOH, 5 kDa in a 1:1 mass ratio. The pegylation reaction was carried out by mixing for 2 h at room temperature in a 7.4 pH environment (10 mM PBS). After this time to the reaction, 0.417–1.67 of trastuzumab per 1 mg of Fe_3_O_4_ was added to synthesize Tmab conjugated NPs. A conjugation reaction with a monoclonal antibody was performed overnight at room temperature as described in our previous paper [30]. Finally, the product was separated by magnetic sedimentation and washed three times with DI water.

### 2.5. Determination of the Number of Trastuzumab Molecules per One Nanoparticle

The average number of Tmab molecules per nanoparticle was determined by a method based on the coupling of radioiodinated trastuzumab ([^131^I]-Tmab) to nanoparticles by using the procedure described before in previous papers [30,31]. Trastuzumab was radioiodinated with ^131^I using the Iodogen method and purified on PD-10 columns. In the next step, 0.417–1.67 nmols of [^131^I]-Tmab was conjugated to NPs-PEG-NHS ester as described above. Nanoparticles conjugated with [^131^I]-Tmab were separated from the reaction mixture, washed up to five times and resuspended in DI water. Radioactivity coupled to NPs to total radioactivity added was measured and compared. The moles of attached [^131^I]-trastuzumab were divided by the moles of used nanoparticles. Moles of used NPs were calculated after ICP-MS (determination of Fe_3_O_4_ amount), TEM analysis (average NPs diameter and in the case of spherical nanoparticles) and a known Fe_3_O_4_ density of 5.2 g/cm^3^.

### 2.6. Stability Studies

The NPs-PEG and NPs-Tmab after synthesis were separated from the reaction mixture and suspended in DI water and 0.9% NaCl solution. The NPs solutions were kept at 4 °C for 14 days to imitate the storage conditions. The aggregation tendency was characterized by the measurements of their hydrodynamic diameter with a DLS method.

### 2.7. In Vitro Cytotoxicity Studies

The cytotoxicity studies were performed by MTS assay for nonradioactive NPs-PEG and NPs-Tmab (0.0–505 µg/mL). SKOV3 cells were cultured and seeded 24 h before the experiment in 96-well plates at a cell density of 2.5 × 10^3^ per well. Then the cells were washed with PBS and treated with rising concentrations of the studied compounds. Compound treated cells were incubated for 18 h at 37 °C, in a 5% CO_2_ atmosphere. After this time the cells were gently washed twice with PBS and fresh medium was added. Then the plates were incubated for 24, 48, and 72 h. After these incubation points, the MTS assay was added to each well, and incubation continued for an additional 2 h at 37 °C, 5% CO_2_ atmosphere in the dark. Lastly, the absorbance was measured at 490 nm with a microplate reader. The results are presented as the cell viability (%) in comparison to the control incubated in the medium only.

### 2.8. Binding Specificity Studies

The receptor binding affinity of NPs-PEG and NPs-Tmab radiobioconjugates was studied in a HER2-positive SKOV-3 cell line according to the procedures described in previous papers [30,31,35]. Tested compounds were incubated in the presence (blocked) or absence (non-blocked) of 100× molar excess of free trastuzumab. Briefly, 2.5 × 10^5^ cells per well were seeded onto 12-well plates 24 h before the experiment and incubated at 37 °C, in a 5% CO_2_ atmosphere. After that, the cells were washed twice with PBS and the studied NPs solutions were added to each well and incubated for 1 h at 4 °C. Next, the medium with unbound nanoparticles was removed and collected, and the cells were washed twice with cold PBS which was collected as well. Finally, the cells were lysed twice with 1 M sodium hydroxide solution. The lysed fraction was collected as well. Collected fractions were measured on an automatic gamma counter.

### 2.9. Internalization Studies

The internalization studies were performed for NPs-Tmab radiobioconjugates in a SKOV-3 cell line. Briefly, the cells were seeded 24 h before the experiment into 6-well plates at the density of 5 × 10^5^ cells per well and cultured at 37 °C, in a 5% CO_2_ atmosphere. Next, the cells were incubated with radiobioconjugate for 1 h at 4 °C. After 1 h, the cell culture medium with unbound NPs was removed, and cells were washed with PBS and incubated with fresh medium at 37 °C for another 1, 12, and 24 h. After each time point, the culturing medium was removed and each well was washed twice with glycine x HCl buffer pH 2.8 and then lysed twice with 1 M sodium hydroxide. Collected fractions were measured on a gamma counter.

### 2.10. Confocal Microscopy Imaging

SKOV-3 cells (10 × 10^3^ per well) were seeded in 12-well plates covered with glass coverslips (ϕ12 mm/#1.5; Thermo Scientific, San Jose, CA, USA). The next day, the medium was removed and the cyanine3 amine labeled NPs-PEG-Cy3 and NPs-Tmab-Cy3 bioconjugates were added. Cells cultured in the presence of a medium were used as a control. After 24 h of incubation, the cells were washed two times with PBS, fixed with 4% PFA for 14 min at 37 °C, permeabilized with a 0.2% (*v*/*v*) PBS solution of Triton X-100 (Sigma) and blocked with a 0.1 % (*v*/*v*) PBS solution of bovine serum albumin (Sigma); each step was separated by washing in 1 mL of PBS twice. Further, cells were stained with a 2.5% PBS solution of Alexa Fluor 488™ Phalloidin (Thermo Fisher Scientific, Waltham, MA, USA). Then, the glass coverslips were mounted on microscope slides with ProLong Diamond Antifade Mountant DAPI (Thermo Fisher Scientific). Dried samples were imaged with a Zeiss LSM 880 confocal laser scanning microscope (Zeiss, Jena, Germany).

### 2.11. Spheroids

The formation of spheroids was initiated by seeding 1 × 10^3^ SKOV-3 cells/well into a 96-well plate with an ultra-low attachment surface (SPL Life Sciences, Gyeonggi, Korea). The spheroids were grown to the size of 70,000–80,000 μm^2^. Next, they were treated with 0–10 MBq per well mL of radiobioconjugates. Some spheroids were additionally treated with magnetic hyperthermia up to 43 °C for about 1 h (field parameters: 404 kHz, 4–19 kA/m). Spheroids were incubated with the radiocompounds for 1 and 18 h, washed multiple times with 100 μL of fresh medium, and finally suspended in a medium that was subsequently replaced every day/every other day. The growth of individual spheroids was measured for up to 9 days after the treatment. The results of spheroid growth or disintegration are presented as % of the surface area in comparison to the surface area at Day 0.

### 2.12. Techniques

The morphology of nanoparticles was examined with a Transmission Electron Microscopy—TEM Zeiss Libra 120 Plus operating at 120 kV (Zeiss, Stuttgart, Germany). The dynamic Light scattering (DLS) method was used to analyze the hydrodynamic size of as-synthesized NPa and bioconjugates being complementary to TEM analysis. The hydrodynamic diameter and zeta potential measurements were carried out in 1 mM PBS pH 7.4 buffer with Zetasizer Nano ZS (Malvern Panalytical, Malvern, Worcestershire, UK). The thermogravimetric analysis—TGA was with performed under a nitrogen atmosphere with TGA Q50 (TA Instruments New Castle, PA, USA). The magnetic hyperthermia—MH experiments were performed with a nanoScale Biomagnetics D5 Series equipment with CAL1 and PC^70^ CoilSets (nB nanoScale Biomagnetics, Zaragoza, Spain). The Specific Absorption Rate -SAR values were estimated using MaNIaC Controller software supplied with D5 series equipment. The absorbances for the MTS assay were measured at 490 nm using an Apollo 11LB913 microplate reader (Berthold, Bad Wildbad, Germany). The radioactivity in binding and internalization assays were measured using Wizard^®^ 2 automatic gamma counter (Perkin Elmer, Waltham, MA, USA) The spheroids observation was made using Primovert microscope with Axiocam 105 color (Zeiss, Jena, Germany) with ZEN 2.3 lite software (Zeiss, Jena, Germany). The confocal imagining was performed using a Zeiss LSM 880 confocal laser scanning microscope (Zeiss, Jena, Germany) with ZEN 2 black edition software. An inductively coupled plasma mass spectrometer with a quadrupole mass analyzer (Nexion 300D, Perkin Elmer, Waltham, MA, USA) and quartz introduction system (Mainhard nebulizer and cyclonic spray chamber) was used for isotope-specific detection (^57^Fe, ^197^Au). Quantification was achieved by external calibration (concentration range: 50–200 µg/L). The iron-to-gold content ratio was calculated by determining the total content of both elements dissolved in and diluted with deionized water samples.

## 3. Results and Discussion 

### 3.1. Synthesis and Characterisation of Fe_3_O_4_@Au Core-Shell Nanoparticles

The core of the nanoparticles was successfully synthesized by the co-precipitation method known and used for previous studies but omitting the holmium doping [30,36,37] and using triamonium citrate as a stabilizing agent. A schematic reaction taking place during the synthesis of iron oxide NPs should be as follows:

Fe^2+^ + 2Fe^3+^ + 8OH^−^ → Fe_3_O_4_ + 4H_2_O (1)

**Figure 1:**
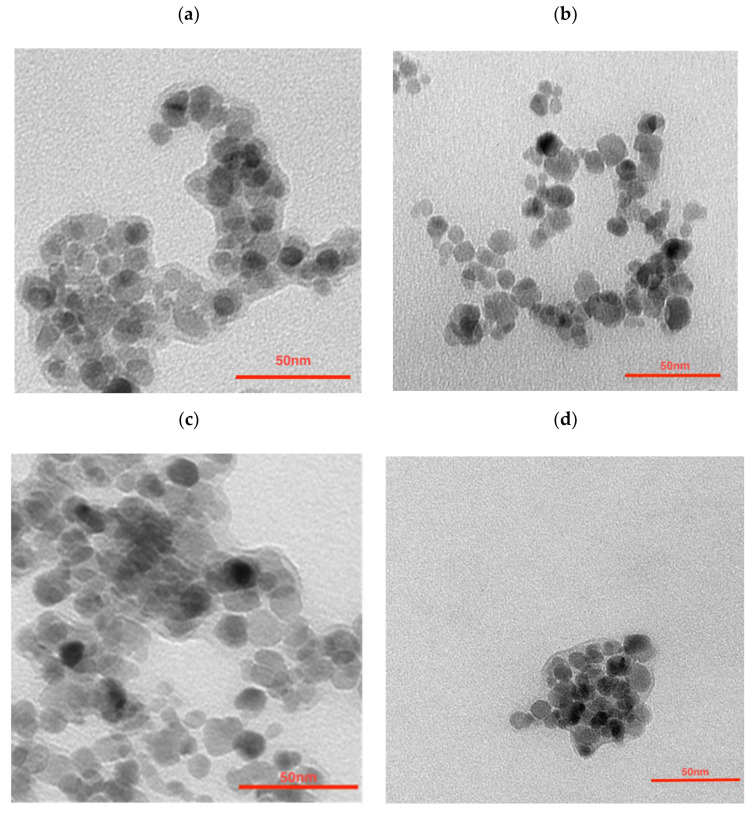
Transmission Electron Microscopy images of (**a**) citrate stabilized NPs, (**b**) NPs-Au, (**c**) NPs-PEG, and (**d**) NPs-Tmab.

To further modify the nanoparticles in order to synthesize the multimodal bioconjugates, a gold coating was made. The efficacy of ^198^Au/Au coating was as seen previously, reaching around a 99% yield. ICP-MS analysis shows that the Fe_3_O_4_:Au ratio was 2.3:1 (*w*/*w*) which is not as different from the ratio used for NPs synthesis—2.5:1. The morphology examination of synthesized nanoparticles was performed with transmission electron microscopy (TEM). As can be seen in Figure 1a the citrate stabilized NPs have well-defined and spherical shapes with a measured average diameter below 10 nm. They are visible as light to dark grey structures with greyish surroundings for which a citrate coating is responsible. Some darker spots visible in this figure are due to the partial overlapping of the iron oxide nanoparticles during the deposition on the TEM support and drying under a vacuum. The gold covers the NPs (Figure 1b) making the NPs darker while some NPs remain uncoated. It can be caused by a heterogeneous gold deposition on part of the nanoparticles only, leaving other nanoferrite cores uncovered. Figure 1c presents the core-shell NPs that were additionally coated with PEG. Larger NPs can be seen but due to the magnetic separation of the product, the presence of sole gold NPs was ruled out—these outliers may be present due to the coating of several aggregated cores. The organic shell is well visible. The trastuzumab is present as the light greyish layer around the whole surface, coating the nanoparticles (Figure 1d).

The DLS measurements were performed in 1 mM phosphate buffer saline pH 7.5 (PBS). The hydrodynamic diameter is displayed as a Z-Average value. As can be seen in Table 1, the size of bare NPs evaluated by DLS was notably larger than that measured by TEM. The main reason for this difference were the solvation layers observed in the DLS technique, whereas in the TEM technique the solid, dry sample is measured, without the solvation layer. Au-coated NPs having less citrate on their surface display a significantly lower diameter: 90.45 ± 2.60 nm compared to 118.4 ± 0.71 nm of citrate-coated NPs. Next, in the case of the NPs-Tmab system, the hydrodynamic size of the bioconjugate is about 123 nm confirming the attachment of the monoclonal antibody onto the NPs surface. It is also significantly larger than that of the nanoparticles modified with PEG alone—92.74 nm. Additionally, the zeta potential was measured to estimate the presence of the functional groups. The zeta potential is a measure of suspension stability: the higher its value (modulus), the more stable the suspension is due to the electrostatic repulsion between the nanoparticles. NPs-citric have a negatively charged surface attributed to the citrate presence on their surface (−48.1 ± 1.0 mV). When the same NPs are coated with gold their surface potential is slightly elevated (−45.4 ± 0.36 mV). The attachment of HS-PEG-NHS ester shifts the zeta potential further towards more positive values (−38.1 ± 0.75 mV). After the conjugation with 149 kDa positively charged protein, the surface potential shifts significantly to −28.9 ± 0.3 mV. However, these surface potential values indicate that the obtained NPs are stable in the studied medium.

The large grayish background visible in Figure 1a is due to the large amounts of citrates used. This background almost disappeared after being used out in the course of the reductive formation of the gold shell (Figure 1b). This greyish “corona” reappeared after pegylation (Figure 1c) and Tmab attachment (Figure 1d).

**Figure 2:**
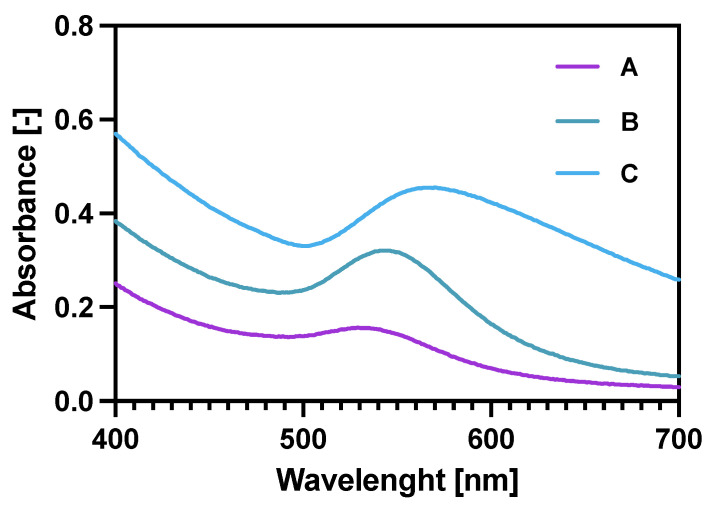
UV-vis spectra for gold coated NPs: A—Fe_3_O_4_: Au 5:1 (*w*/*w*); B—Fe_3_O_4_: Au 2.5:1 (*w*/*w*); C—Fe_3_O_4_: Au 1.25:1 (*w*/*w*).

Checking the UV-vis spectra (Figure 2) for the core-shell NPs was another (after ICP-MS and TGA) way of proving the gold reduction on the NPs. Additionally, it was also proven that increasing the Au:Fe_3_O_4_ ratio (spectra A–C) significantly broadens the characteristic Au plasmon peak, shifting it also towards longer wavelengths. These two features prove the increased amount of Au deposited on the magnetic core [38,39]. Finally, the coating 2.5:1 was chosen for future experiments as it provides a sufficient specific radioactivity of 100 MBq/mg of Fe_3_O_4_ and binding specificity in comparison to 5:1 and 1.25:1 bioconjugates synthesized in the same manner.

### 3.2. Thermogravimetry Analysis

**Figure 3:**
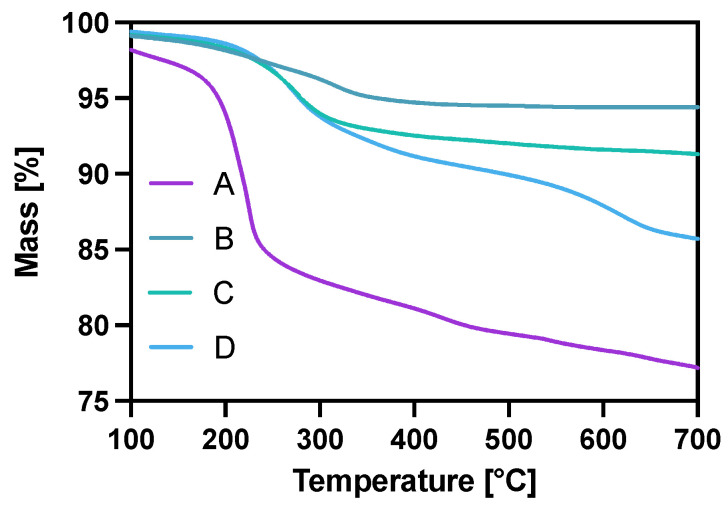
Thermograms of A—NPs-citric, B—NPs-Au, C—NPs-PEG, D—NPs-Tmab.

The modification of Fe_3_O_4_ in each step was also controlled with thermogravimetric analysis (TGA) under the ambient atmosphere in the temperature range from room temperature to 700 °C, with a heating rate of 10 °C/min. The gradual mass loss while heating can be related to the decomposition of organic compounds modifying the surface of nanoparticles. As can be seen in Figure 3, the mass loss for the citrate-modified core is ca. 22% with the water-related loss of 2% before reaching the 100 °C degree mark. After gold coating and surface citrate consumption during the Au^3+^ reduction, the mass loss for NPs-Au (B) is about 5.6%. The PEG coating (C) contributed to a mass decrease of about 9.7% with heating, of which 4.1% may be attributed to the PEG coverage alone (5.6% remaining citrates). In the case of the NPs-Tmab (D) sample, we see a large decrease in mass due to—the decomposition of the attached protein and linker agent (PEG) indicating the successful formation of the bioconjugate.

### 3.3. Estimation the Number of Attached Trastuzumab per One Magnetite Nanoparticle

To estimate the number of Tmab molecules attached to one nanoparticle, the synthesis of bioconjugate using [^131^I]-labeled Tmab was performed (Section 2). Based on the obtained results, the amount of [^131^I]-Tmab per mg of Fe_3_O_4_ core was found to be 107.0 ± 1.5 μg with a coupling efficiency of 81.7 ± 7.8%. The number of Tmab particles per one NPs was calculated as described in previous papers: each nanoparticle is spherical, has an average diameter of 12 nm and the magnetite density is 5.2 g/cm^3^ (we neglected the mass of attached Au). The obtained results indicate that approximately two to five trastuzumab molecules were coupled to one nanoparticle, depending on the amount of monoclonal antibody used for synthesis. In our previous experiments, we proved that two trastuzumab molecules coupled to one core-shell nanoparticle allowed achieving a binding specificity of 5.43% [30].

### 3.4. Hyperthermia Studies

**Figure 4:**
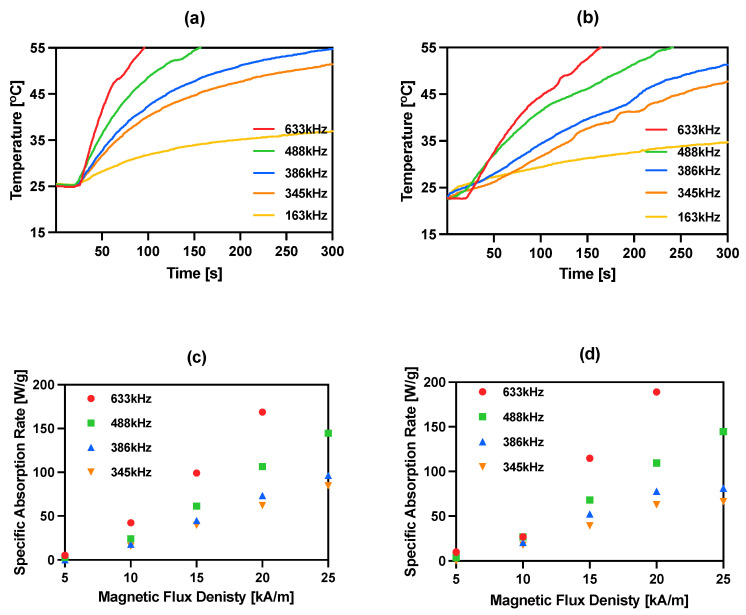
Heating of bioconjugates in various ranges of frequency of magnetic field: (**a**) NPs-PEG 23 kA/m; (**b**) NPs-Tmab 23 kA/m and dependence of SAR for various frequencies of the magnetic field in the function of the amplitude of magnetic field: (**c**) NPs-PEG; (**d**) NPs-Tmab.

The magnetic properties of iron oxide nanoparticles conjugated with different molecules, including trastuzumab, have been studied by our group during previously reported experiments [15,30,36,40,41]. In this work, magnetic hyperthermia studies were performed for NPs-PEG and NPs-Tmab radiobioconjugates. The aqueous suspension of 8 mg/mL of Fe_3_O_4_ was placed in a thermostated copper coil and the temperature changes were recorded under the effect of the alternating magnetic field in the frequency range 163–633 kHz and amplitude from 5 to 25 kA/m. The measurements were performed until the temperature reached 55 °C or for 5 min. As it was proven many times before for similar suspensions, the increase in AMF frequency or amplitude generates higher heating rates. For practical reasons, only the frequencies above 345 kHz should be considered in further experiments, as they allow reaching the desired hyperthermic temperature values of 42–45 °C in a short time, and therefore, the frequency of 163 kHz was ruled out for further applications. For the obtained bioconjugates, the SAR values can reach as high as 189.1 W/g for NPs-Tmab and 168.8 W/g for NPs-PEG when a 633 kHz and 20 kA/m AMF are applied. The heating rate measurements and SAR dependence in relation to the frequency and amplitude are displayed in Figure 4. The SAR values were calculated using the ZaR subprogram of MaNIaC 1.0 Software using the equation reported before [30]. From a biomedical point of view, the frequency and amplitude quotient should be kept below the so-called Brezovich limit for hyperthermia therapy—4.85 × 10^8^ AHz/m [42,43].

The current literature reports a large range of SAR values, starting from 85 W/g for hydroxyapatite-coated Fe_3_O_4_ [44], 248 W/g for PEG-modified NPs conjugated with folic acid as a targeting agent [45] and 1018 W/g for Mn,Zn-doped iron oxide [46]. We found our nanoparticles fit into the trend of previously reported achievements [15,40,41]. Obtained NPs radiobioconjugates are effective heat mediators in the moderate (345–386 kHz) studied frequencies and can achieve temperatures over 41 °C desired for in vitro studies. These low SAR values that are below 1000 W/g result, however, in the need for the application of higher bioconjugates concentrations that are >5 mg/mL.

### 3.5. Stability Studies

**Figure 5:**
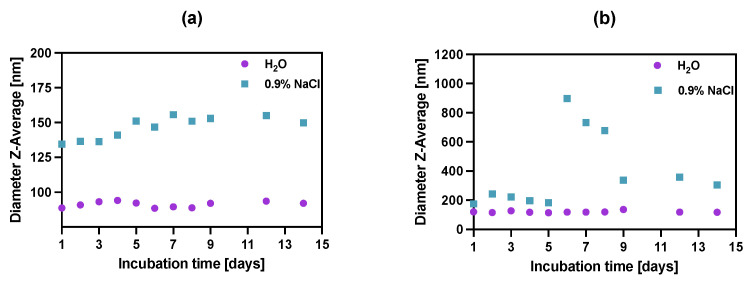
Stability studies of (**a**) NPs-PEG, (**b**) NPs-Tmab in water DI and 0.9% NaCl. Data are expressed as mean value ± SD (*n* = 3). The error bars are the size of symbols used.

To study the stability of synthesized bioconjugates, the incubation in water DI and saline (0.9% NaCl) was carried out over two weeks (Figure 5) by measuring the dynamic light scattering of the suspension. Unfortunately, in the case of biological media (e.g., growth medium or human serum), the use of dynamic light scattering is inappropriate due to the presence of high amounts of proteins that also scatter light, affecting the DLS measurements.

The stability of bioconjugates was determined by DLS in two storage solutions, through the measurements of hydrodynamic diameter over the period of 14 days. The tested conjugates showed great size stability for up to 5 days, after which the NPs-Tmab bioconjugate suspensions started to aggregate in 0.9% NaCl solution increasing their size more than four times. This was followed by a further decrease in their size to 150% of the starting diameter over the next several days. This may be due to the known phenomenon of “salting out”: increasing the ionic strength precipitates the colloid, destabilizing both surface hydration (lipophilic colloids) and compensating for the electrostatic repulsion (lyophobic colloids). A further decrease in the hydrodynamic diameter could be attributed to the degradation or dissolution of NPs aggregates. It is worth noting the NPs-PEGs have better stability in the water as well as in 0.9% NaCl solution, as compared to NPs-Tmab, with a slight size increase in the latter by ca. 12% of the original size at Day 1. The bioconjugate NPs-Tmab also showed great stability in water solution for an extended period.

### 3.6. In Vitro Cytotoxicity Results

**Figure 6:**
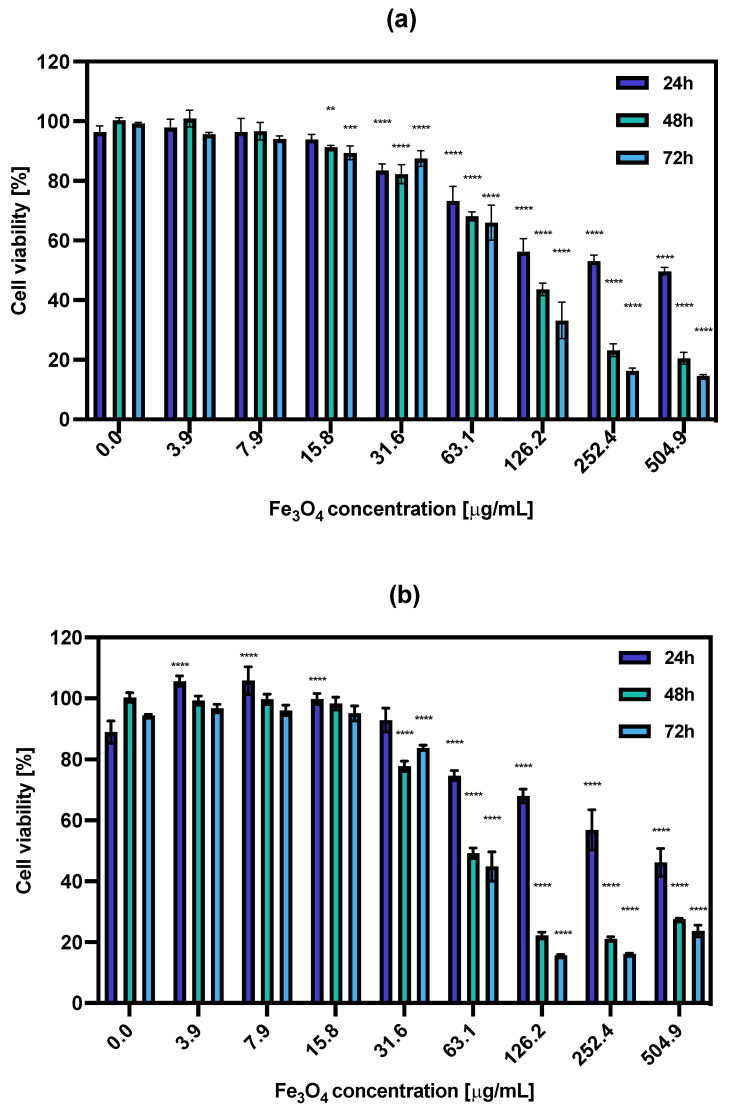
Cell viability after treatment with different concentrations of (**a**) NPs-PEG, (**b**) NPs-Tmab. SKOV-3 were incubated for 24, 48 and 72 h. The results are expressed as a percentage of control cells. Data are expressed as mean value ± SD (*n* = 3). Statistical confidence was considered if *p* < 0.01 (**), *p* < 0.001 (***), *p* < 0.0001 (****).

The cytotoxic effect of non-radioactive NPs-PEG and NPs-Tmab was tested on the SKOV-3 cell line in relation to their treatment with increasing concentrations (0.0–504.9 μg/mL) of the studied compounds for 18 h. Then, the medium was removed and the cells were washed with PBS and supplemented with fresh medium followed by further incubation for 24, 48, and 72 h. The cell viability was assessed with an MTS assay (Figure 6). The obtained results show that the obtained nonradioactive radiobioconjugates are non-toxic to cells in concentrations up to 31.6 μg/mL as the cell viability decreased roughly by ca. 20% in some cases. However, increasing the concentrations resulted in enhanced cytotoxicity toward cancer cells. This positive result was surprising because the results obtained previously for holmium doped core-shell NPs [30] showed no such effect. The cytotoxicity increased in a concentration and time-dependent manner, starting at Fe_3_O_4_ concentration (NPs-PEG) at 63.1 μg/mL (73.3 ± 4.9% after 24 h to 66.0 ± 5.9% after 72 h) and reaching as low as 14.6 ± 0.4% for 72 h incubation with 504.9 μg/mL.

The calculated IC_50_ values ranged from 326.1 μg/mL for 24 h incubation with NPs-PEG which was followed by 110.5 μg/mL and 91.08 μg/mL for 48 and 72 h, respectively. For the NPs-Tmab the calculated IC_50_ was shown to be 353.9 μg/mL for 24 h and 73.09 and 63.04 μg/mL for 48 and 72 h incubation times. This indicates that the cytotoxicity of NPs-PEG was slightly lower than that obtained for NPs-Tmab for concentrations higher than 126.2 μg/mL and longer incubation times (48 and 72 h). This may be attributed to the uptake of Tmab-modified nanoparticles which could be a possible cause for increased cytotoxicity. Generally, the dissolution of some uncoated or partially coated nanoparticles over time in a growth medium could impact the increased cytotoxicity of nanoparticles. This dissolution could increase the concentration of iron (II) and (III) ions in the solution which is attributed to cytotoxicity changing the medium’s electrolytic composition. This can be seen especially for the high concentration of nanoparticles used in this study.

### 3.7. Spheroid Disintegration Studies

**Figure 7:**
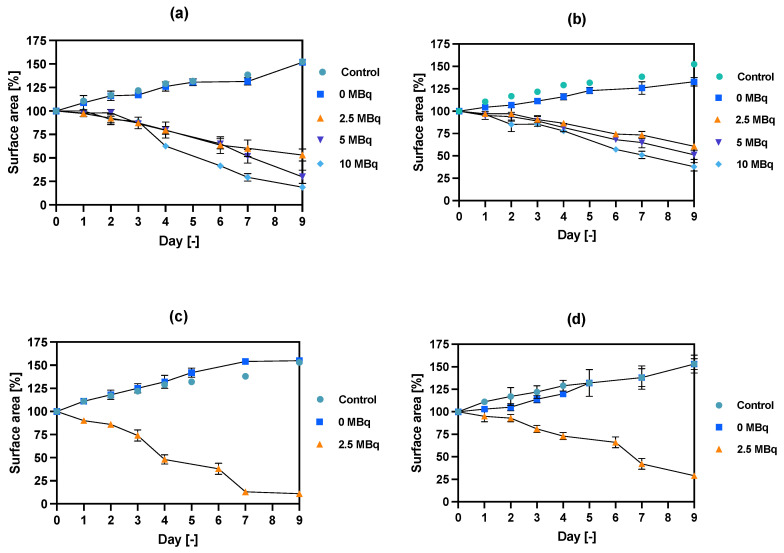
Spheroid growth over time treated with different activities of NPs bioconjugates: (**a**) radioactive NPs-PEG, 1 h incubation; (**b**) radioactive NPs-Tmab, 1 h incubation; (**c**) radioactive NPs-PEG, 18 h incubation; (**d**) radioactive NPs-Tmab, 18 h incubation. For some experimental data the error bars are the size of the solid symbols used. Data are expressed as mean value ± SD (*n* = 3).

**Figure 8:**
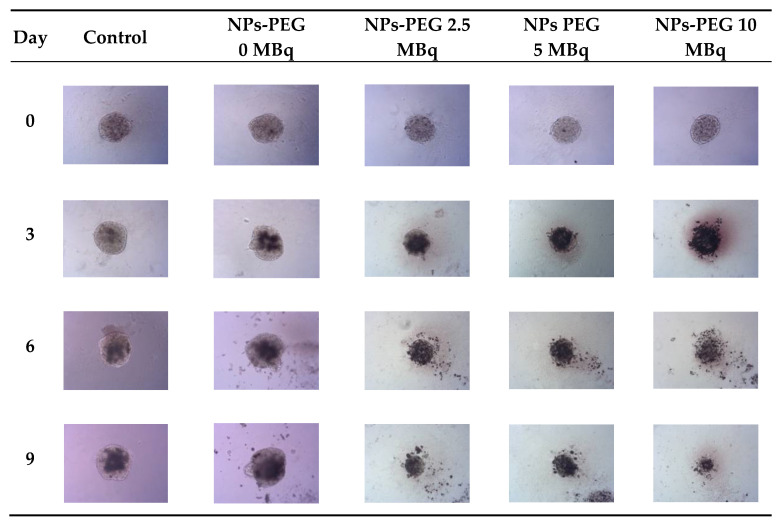
Microscope images of representative spheroids. Images made for spheroids incubated for 1 h with radioactive NPs-PEG on the day of treatment (Day 0) and on Days 3, 6 and 9 after treatment. Images were acquired with Primovert microscope equipped with Axiocam 105 color (Zeiss, Jena, Germany) with ZEN 2.3 lite software (Zeiss, Jena, Germany), 5× magnification.

**Figure 9:**
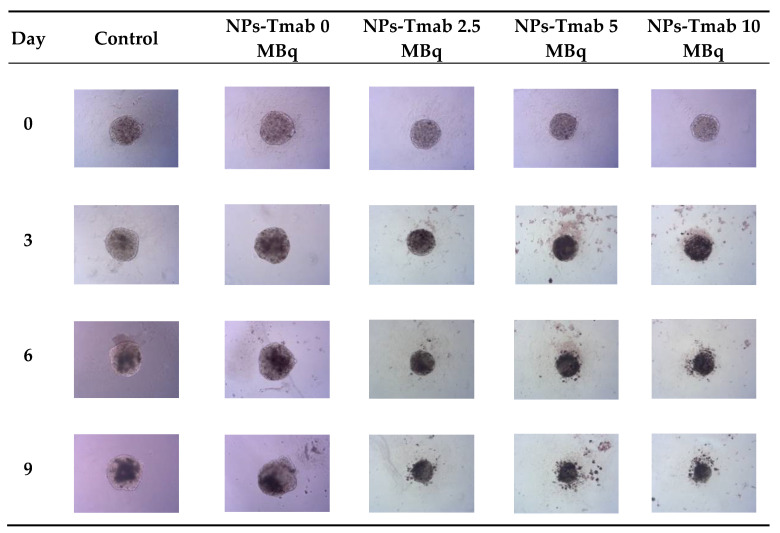
Microscope images of representative spheroids. Images made for spheroids incubated for 1 h with radioactive NPs-Tmab on the day of treatment (Day 0) and on Days 3, 6, and 9 after treatment. Images were acquired with Primovert microscope equipped with Axiocam 105 color (Zeiss, Jena, Germany) with ZEN 2.3 lite software (Zeiss, Jena, Germany), 5× magnification.

The spheroids (SKOV-3 cells) for the reported experiments were cultured for at least 7 days to obtain well-defined spherical structures of a surface area of 70–80 × 10^3^ μm^2^. The culture medium was exchanged every other day during the culturing process. Next, the spheroids were treated with NPs radiobioconjugate suspensions with activity doses of 2.5, 5.0 and 10 MBq per well, corresponding to 25, 50 and 100 µg/mL of Fe_3_O_4_. Spheroids were treated with specific doses for 1 and 18 h, after which the NPs were removed by multiple washings. The untreated spheroids were used as a control. The surface area was then measured every day or every other day with the help of a microscope. Spheroid growth over time after treatment and pictures of the representative spheroids, for illustrative purposes, are displayed in Figure 7, Figure 8 and Figure 9, respectively. In the case of Figure 8 and Figure 9, the spheroid images were taken under the same 5× magnification (Axiocam 105 color adapter 0.5× with 10× objective).

In comparison to the 2D cytotoxicity studies, the nanoparticles show no toxicity on spheroids. In the case of both conjugates: NPs-PEG (incubated for 1 and 18 h), and NPs-Tmab (incubated for 1 h), the spheroid growth is comparable to the control—growth to 155 ± 6% of the size at Day 0. Only spheroids treated with NPs-Tmab for 1 h show smaller overall growth in comparison to control of about 19.6%. After incubation with radioactive conjugates the spheroids reduce their area and begin to degrade, starting on day 3 for 10 MBq dose, and day 5–6 for lower 2.5–5.0 MBq doses. In all cases, the spheroid growth is inhibited. The overall differences in the degradation were higher for NPs-PEG where substantial degradation up to 18.9 ± 2.5% was achieved for the highest dose of 10 MBq followed by 29.9 ± 7.1% for 5.0 MBq and 53 ± 6.4 for 2.5 MBq. Prolonged incubation (18 h) with lower doses of 2.5 MBq NPs-PEG showed similar degradation to treatment with 10 MBq for 1 h (NPs-PEG)—11.0 ± 3%. In the case of NPs-Tmab the spheroid surface area was reduced to 60.8 ± 1.0 %, 51.3 ± 5.1 % and 37.8 ± 4.7% for 2.5, 5.0 and 10 MBq, respectively. Again the prolonged incubation with a lower dose proved to have a similar impact on the degradation as the 10 MBq 1 h treatment. The low differences in toxicity between NPs-PEG and NPs-Tmab are worth mentioning. It is related to the fact that in the case of nanoparticles labeled with ^198^Au, emitted β-radiation has a range large enough to kill cells without binding to the receptor. Therefore, conjugation with a targeting vector (Tmab) has no effect on cytotoxicity. This was due to the fact that NPs-Tmab had lower stability in the growth medium which was surprisingly attributed to greater efficiency in the removal of aggregated NPs. The NPs-PEG, however, were more stable and well dispersed which influenced the washings, thus leaving the higher dose in the wells in comparison to NPs-Tmab. This difference nevertheless was small, ca. 5–10% of added radioactivity.

### 3.8. Binding Affinity

**Figure 10:**
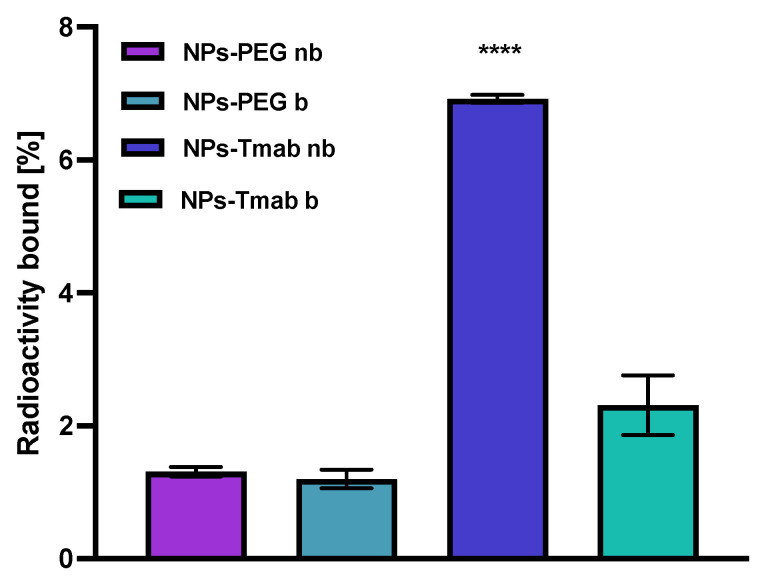
Specificity of binding on SKOV-3 (HER2+) of NPs-PEG and NPs-Tmab; nb—nonblocked, b—blocked with 100 molar excess of free Tmab. Data are expressed as mean value ± SD (*n* = 3). Statistical confidence was considered if *p* < 0.0001 (****).

The affinity of NPs-PEG and NPs-Tmab radiobioconjugates for HER2 receptors was examined in the SKOV-3 cell line. The tested compounds were incubated in the presence (blocked) or absence (nonblocked) of a 100-fold molar excess of free trastuzumab. The results are presented in Figure 10. Due to previous findings, we did not conduct binding experiments in HER2 negative cell lines (e.g., MDA-MB-231) because Tmab-modified modified core-shell bioconjugates show no specific activity toward HER2 negative cancer cells [30,31]. In this paper, we limited experiments to one-line specificity testing with blocking with excess trastuzumab.

In case of NPs-PEG radiobioconjugate, as was expected, there is no significant difference between the results obtained with or without blocking with Tmab excess (*p* > 0.05). For the NPs-Tmab bioconjugate, there is a significant difference between the blocked and nonblocked cell lines with the specific binding of 4.61% (*p* < 0.0001) which is fairly similar to the specific binding determined in the previous paper [30] for similar NPs/Tmab based bioconjugates [31]. There is also a significant difference between the competitive binding of NPs-Tmab and NPs-PEG with blocking 1.11% (*p* < 0.0001) and without blocking 1.00% (*p* < 0.0001). The obtained radiobioconjugates showed reduced background binding or sedimentation, which may be attributed to modifications in the synthetic procedure reported previously [30]. An increase in the reaction time between NPs-Au and HS-PEG-COOH could be attributed to an increase in the amount of PEG molecules per one magnetic nanoparticle which reduced the sedimentation and sorption of NPs to the cell surface thus increasing its stability in the growth medium. In effect, the nanoparticles could be removed easier during the rinsing step and that resulted in a lower binding background below 2%.

### 3.9. Internalization Studies 

**Figure 11:**
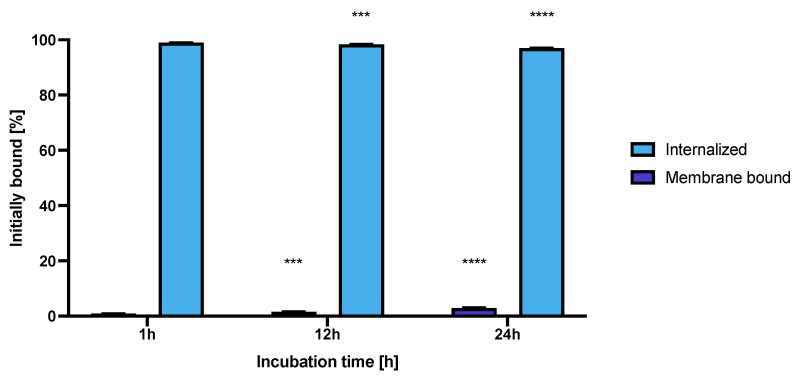
Internalization of NPs-Tmab on SKOV-3 (HER2+) observed after 1, 12 and 24 h of incubation with studied compounds. Data are expressed as mean value ± SD (*n* = 3). Statistical confidence was considered if *p* < 0.001 (***), *p* < 0.0001 (****).

The internalization of the NPs-Tmab radiobioconjugate in SKOV-3 cells was tested at three incubation time points at 37 °C. The obtained results are presented in Figure 11.

**Figure 12:**
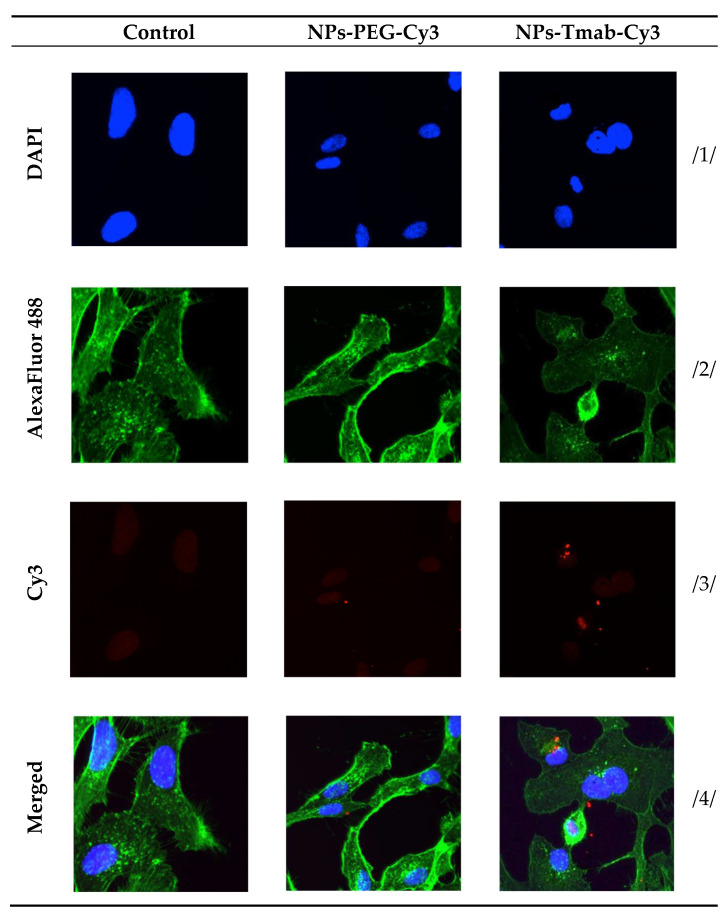
Internalization of the NPs-PEG-Cy3 and NPs-Tmab-Cy3 bioconjugates by SKOV-3 cells determined by confocal microscopy. Cells treated and untreated (control) are shown as a reference. Fluorescence signals present: /1/ blue, nuclei localization; /2/ green, cytoskeleton; /3/ red, Cy3 modified NPs; /4/ merged photos. Magnification 20× with Zeiss LSM 880 confocal laser scanning microscope (Zeiss, Jena, Germany) with ZEN 2 black edition software.

The high and quick internalization of free trastuzumab is well known [47]. Our bioconjugate internalizes quickly after just 1 h of incubation to up to 99.03 ± 0.07%. Surprisingly over the period of 24 h incubation the radiobioconjugate internalization decreased significantly to 98.39 ± 0.12% for 12 h (*p* < 0.001) and 97.00 ± 0.20% (*p* < 0.0001). The obtained results indicate that radiobioconjugates were rapidly internalized, but in time they were exerted from the cells as indicated by the increasing membrane binding. Our results apparently agree with the previous findings for barium ferrite radium-223-doped NPs, which also had over 90% internalization levels [31]. The obtained radiometric internalization results were verified by the confocal imaging of NPs modified additionally with a cyanine 3 fluorescent dye. The SKOV-3 cells were incubated with the studied compounds for 24 h and untreated cells were used as the control. The obtained pictures are displayed in Figure 12. The small red dots indicate the NPs presence (Figure 3b,c and Figure 12). Merged signals (Figure 4b,c and Figure 12) show that only NPs-Tmab-Cy3 penetrated the cell membrane and show intracellular uptake; those unmodified with monoclonal antibody (NPs-PEG-Cy3) are located outside the SKOV-3 cells near the cellular membrane which may be attributed to the low unspecific binding of NPs-PEG shown before. The absence of a targeting vector lowers the internalization abilities of nanoparticles. These results are comparable with our previous findings [31,48,49,50]. The obtained results are in good relation with binding and internalization studies. PEG-modified bioconjugates are localized near the cell membrane in low amounts, while those modified with monoclonal antibodies (Tmab) are localized mainly inside the SKOV-3 cells.

### 3.10. Hyperthermia and Radiation Influence on Spheroid Growth

**Figure 13:**
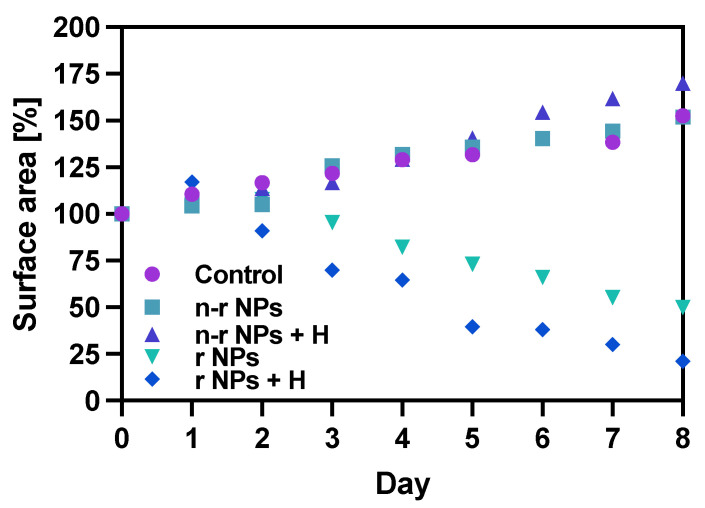
Spheroid growth over time treated with NPs bioconjugate or untreated (control): n-r—nonradioactive, r—radioactive, H—hyperthermia. Data are expressed as mean value ± SD (*n* = 3).

**Figure 14:**
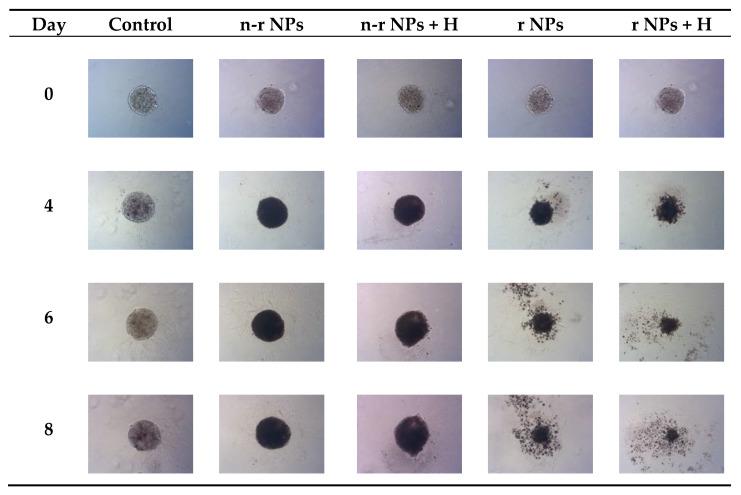
Microscope images of representative spheroids. Images made for spheroids on the day of treatment (Day 0) and on Days 4, 6 and 8 after treatment: n-r—nonradioactive, r—radioactive, H—hyperthermia.

The spheroids were grown in the same way as described above and treated with a dose of bioconjugate of about 7.5 MBq per well and Fe_3_O_4_ concentration of 8 mg/mL to achieve a temperature rise of 5–6 °C. The results of spheroid surface area measurements and representative photos of the spheroid are presented in Figure 13 and Figure 14, respectively.

The obtained results are in coherence with those presented above regarding the cytotoxicity of “cold” nanoparticles towards spheroids. Surprisingly, the spheroids treated with NPs and hyperthermia show increased growth compared to the control (about 17.6% higher). However, according to the statistical analysis, this was not a significant difference (*p* > 0.05). The influence of hyperthermia with radionuclide treatment was, however, clearly visible in this study. Not only did hyperthermia initiate the faster degradation of spheroids (starting on the first day after the treatment vs. the second) but also had a greater overall effect on the surface area of 28.9% (*p* < 0.001) which is over two times lower than spheroids treated with radioactive bioconjugate alone. The images taken (for the illustrative purpose) during the observed 8^−^day degradation are presented in Figure 14. 

## 4. Conclusions

The iron oxide superparamagnetic nanoparticles were covered successfully with radioactive gold-198, as proved by three separate methods: UV-*vis* spectrophotometry, radiochemically, and ICP-MS analysis. Further modifications regarding linker and biomolecule conjugations were proven by two separate methods as well. We showed that synthesized NPs bioconjugates have good stability in water and in 0.9% NaCl solution for a period of 5 days. The obtained radiobioconjugates show good binding specificity and internalization in accordance with our previous studies [30]. The heating capabilities of NPs were retained and are still promising for further biomedical applications regarding in vivo studies. Three-dimensional culture studies proved that applying hyperthermia simultaneously with radionuclide treatment generates more than two times improved results in terms of the spheroid degradation process. Iron oxide NPs allow to generate heat in AMF, are biodegradable, and can be kept in cancer tissue using a constant magnetic field. The use of trastuzumab as a targeting vector yielded the creation of a radiobioconjugate of good specific binding to HER2-positive cancer cells. Obtained nanoparticles have good heating capabilities and can be synthesized with high specific activity and high radiolabeling yield. The usage of gold as coating paved the way for the application of thiol bifunctional linkers which have broad applications in biomedicine and are easy to use in various bioconjugations. The obtained results show that the obtained radiobioconjugates could be potentially used in the treatment of HER2-positive small cancers, especially breast and ovarian early-stage cancers. The application of hyperthermia and radionuclide therapy in a single nanoparticle yielded two times better results in spheroid degradation compared to separate treatments in 3D cell structures. It is also worth noting that an additional advantage of the proposed Fe_3_O_4_@^198^Au-Tmab radiobioconjugate is the simple ability to control the therapeutic process using NMR imaging as shown in the paper of Rajkumar S. et al. [51].

## Figures and Tables

**Table 1:** Values of hydrodynamic diameter, zeta potential and PI of NPs measured in PBS.

	Hydrodynamic Diameter [nm]	Zeta Potential [mV]	Polydispersity Index [-]
NPs-citric	118.4 ± 0.7	−48.1 ± 1.0	0.232 ± 0.007
NPs-Au	90.4 ± 2.6	−45.4 ± 0.36	0.225 ± 0.07
NPs-PEG	92.7 ± 1.6	−38.1 ± 0.75	0.215 ± 0.011
NPs-Tmab	123.0 ± 3.6	−28.9 ± 0.3	0.208 ± 0.016

## Data Availability

All other relevant data of this study are available from the corresponding authors upon reasonable request.
